# Comparison of mineral precipitation, elemental release, pH change and cytotoxicity of calcium-silicate cements and an experimental resin-modified glass ionomer cement containing bioactive glass

**DOI:** 10.2340/biid.v13.45796

**Published:** 2026-04-28

**Authors:** Wisitsin Potiprapanpong, Parichart Naruphontjirakul, Naruporn Monmaturapoj, Siriporn Tanodekaew, Somruethai Channasanon, Arnit Toneluck, Somying Patntirapong, Piyaphong Panpisut

**Affiliations:** a Faculty of Dentistry, Thammasat University, Pathum Thani, Thailand; b Biological Engineering Program, Faculty of Engineering, King Mongkut’s University of Technology Thonburi, Bangkok, Thailand; c National Metal and Materials Technology Center (MTEC), National Science and Technology Development Agency, Pathum Thani, Thailand

**Keywords:** resin-modified glass ionomer cement, bioactive glass, dentin remineralisation, pH change, toxicity, Ca-Si cement

## Abstract

**Introduction:**

Resin-modified glass ionomer cements (RMGICs) exhibit lower remineralising potential than calcium-silicate (Ca-Si) cements. This study aimed to prepare an experimental RMGIC incorporating Sr/F-bioactive glass nanoparticles (EXP) to enhance remineralisation on demineralised dentine. The experimental material was compared with commercial Ca-Si cements (RetroMTA [MTA], Biodentine [BDT], Theracal LC [TC]) and a commercial RMGIC (Vitrebond [VB]).

**Materials and methods:**

Demineralised dentine specimens were attached to discs of each material and immersed in simulated body fluid for up to 4 weeks. Mineral precipitation was assessed using an Attenuated Total Reflection – Fourier Transform Infrared Spectroscopy (ATR-FTIR) and scanning electron microscopy-energy-dispersive X-ray spectroscopy analyses. Remineralisation was quantified as the mineral-to-collagen ratio, calculated from the phosphate FTIR peak height at 1024 cm^-1^ relative to the amide I peak at 1636 cm^-1^ (*n* = 5). Changes in pH and elemental release (Na, Al, Si, P, Ca and Sr) from materials immersed in deionised water over 4 weeks were also evaluated (*n* = 3). Indirect cytotoxicity of material extracts on human dental pulp stem cells was assessed using the 3-(4,5 dimethylthiazol-2-yl)-2,5-di-phenyltetrazolium bromide assay (MTT) assay (n=4). Statistical analysis was performed using one-way analysis of variance (ANOVA) followed by Tukey’s Honestly Significant Difference test.

**Results:**

The highest increase in the mineral-to-collagen ratio was observed with MTA (78%), which was significantly higher than that of VB (−28%) and EXP (−33%). Scanning electron microscopy analysis demonstrated mineral precipitation with MTA, BDT and TC but not with VB or EXP. Additionally, MTA, BDT and TC produced the highest alkalinisation of the storage solution (pH~12). EXP exhibited the release of multiple ions (Na, Al, Si, P, Ca and Sr). Although MTA showed the highest cell viability, all materials demonstrated cell viability exceeding 70%.

**Conclusion:**

The experimental RMGIC containing Sr/F-bioactive glass nanoparticles exhibited inferior remineralising potential compared with Ca-Si cements. However, it promoted the release of multiple essential ions.

KEY MESSAGES

1. An experimental resin-modified glass ionomer cement (RMGIC) containing Sr/F bioactive glass nanoparticles released multiple ions and showed acceptable *in vitro* cytocompatibility.
2. The experimental RMGIC in the current study exhibited lower remineralising actions on demineralised dentine and pH elevation compared with Ca-Si cements.

## Introduction

Dental caries remains one of the most prevalent preventable chronic diseases worldwide [1]. With continued global population growth, the number of individuals affected by active caries in permanent and primary teeth is projected to reach 2.26 and 0.52 billion, respectively, by 2050 [2]. Contemporary management of deep, active, cavitated carious lesions increasingly favors minimally invasive approaches, such as selective caries removal [3]. This strategy involves the complete removal of carious tissue at the lesion periphery while preserving partially demineralised dentine near the pulp, thereby reducing the risk of pulp exposure [4]. However, bonding to caries-affected dentine remains challenging, as conventional adhesive systems often exhibit reduced bond strength due to incomplete resin monomer infiltration into the altered dentine substrate [5]. This compromised adhesion may increase susceptibility to hydrolytic and enzymatic degradation, ultimately undermining the long-term durability of restorations [6, 7].

The placement of a liner over deep caries was expected to help maintain the bonding performance of the resin composite to caries-affected dentine [8]. However, there is currently no consensus on the optimal liner protocols, which remains an area requiring high-quality randomised clinical trials [9]. Recent systematic reviews have suggested that the use of various liners, such as calcium-silicate (Ca-Si) cements and glass ionomer cements, does not significantly influence clinical outcomes when an effective coronal seal is achieved [10, 11]. Despite this, liner placement may still provide protection against mechanical or chemical irritation of the pulp in deep cavities [10, 12, 13].

Resin-modified glass ionomer cements (RMGICs) are among the most commonly used liner materials in deep cavities restored with resin composites. Their advantages include ease of handling, light-activated polymerisation and reliable bonding to resin composites [14]. However, conventional RMGICs contain relatively high concentrations of low-molecular-weight monomers, such as 2-hydroxyethyl methacrylate (HEMA), which raise concerns regarding cytotoxicity [15]. HEMA has been shown to induce oxidative stress, DNA damage, cell-cycle arrest and apoptosis *in vitro* [16]. Additionally, the release of unreacted monomers may also contribute to environmental contamination from dental waste materials [17].

To overcome these limitations, a low-HEMA RMGIC formulation containing only 5 wt% HEMA was previously developed [18]. These experimental RMGICs demonstrated satisfactory physical properties, ion-releasing capability and reduced cytotoxicity compared with commercial RMGICs [18–20]. Nevertheless, RMGICs generally exhibit inferior remineralisation potential compared with Ca-Si cements [18–20]. Incorporation of bioactive glass (BAGs) into glass ionomer-based materials has been proposed as a strategy to enhance bioactivity and remineralisation [21]. Bioactive glasses release biologically relevant ions, such as calcium, phosphate and silicate, which promote supersaturation and facilitate mineral deposition within demineralised dentinal collagen fibrils [21, 22]. In particular, strontium and fluoride released from BAGs may synergistically promote the formation of strontium-substituted hydroxyapatite and fluorohydroxyapatite, potentially contributing to caries prevention [23]. A previously developed experimental RMGIC containing Sr/F-bioactive glass nanoparticles (Sr/F-BAGs) also demonstrated enhanced fluoride release (136 ppm) compared with a commercial RMGIC (88 ppm) [18].

However, the remineralising potential of this low-HEMA experimental RMGIC incorporating Sr/F-BAGs has not yet been investigated. Therefore, the aim of the present study was to evaluate the ability of this experimental RMGIC to promote mineral precipitation on demineralised dentine, in comparison with commercial Ca-Si cements and a commercial RMGIC. In addition, changes in pH, elemental release and cytotoxicity towards human dental pulp stem cells were assessed. The null hypothesis was that there would be no significant differences between the experimental RMGIC and commercial materials in terms of mineral precipitation, pH change, elemental release and cytotoxicity.

## Materials and methods

**Figure 1:**
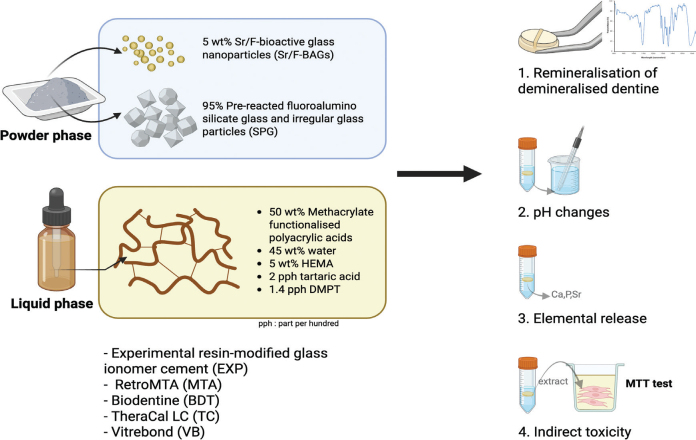
Schematic illustration of the experimental design of the present study. Created in BioRender. Panpisut, P. (2026) https://BioRender.com/w35d26e

The formulation of the experimental resin‑modified glass ionomer cement (EXP) used in this study was selected based on satisfactory physical performance reported in the previous study [18]. Commercial materials used for comparison included RetroMTA (MTA; BioMTA, Soul, South Korea), Biodentine (BDT; Septodont, Saint-Maur-des-Fossés, France), Theracal LC (TC; Bisco, Schaumburg, IL, USA) and Vitrebond (VB; 3M, Saint Paul, MN, USA). The compositions of the experimental and commercial materials are presented in Table 1 and Figure 1.

**Table 1:** Composition of the experimental resin-modified glass ionomer cement (EXP), commercial calcium-silicate (Ca-Si) cements and the commercial resin-modified glass ionomer cement.

Materials (Abbreviation)	Composition	Suppliers	Instruction
Experimental RMGIC (EXP)	Powder: 95% fluoroaluminosilicate glass (SPG), 5% Sr/F-BAGs Liquid: polyacid (50 wt%), HEMA (5 wt%), water (45 wt%), tartaric acid (2 parts per hundred), diphenyl (2,4,6-trimethylbenzoyl)phosphine oxide (1.5 part per hundred), N,N-dimethylaminoethyl methacrylate (1.4 part per hundred)	-	Mix powder and liquid at a 1.5:1 mass ratio by hand for 15 seconds; light-cured for 20 seconds.
RetroMTA (MTA)	Calcium carbonate 60~80%, silicon dioxide 5~15%, aluminium oxide 5~10%, zirconium dioxide 20~30%	BioMTA, Seoul, South Korea	Use 0.3 g powder with three drops of liquid and mixed by hand for 20–40 seconds
Biodentine (BDT)	Powder: Tricalcium silicate (main core material), dicalcium silicate (second main core material), calcium carbonate and oxide (filler), iron oxide (shade), zirconium oxide (radiopacifier) Liquid: Calcium chloride (accelerator), dihydrate (hydrosoluble polymer), water	Septodont, Saint-Maur-des-Fossés, France	Add 5 drops of liquid to the capsule and then mix with an amalgamator for 30 seconds.
Theracal LC (TC)	Calcium-silicate cement (30–50%), polyethene glycol dimethacrylate (10–30%), barium zirconate powder (1–10%)	Bisco, Schaumburg, IL, USA	Inject from the syringe, light-cured for 20 seconds
Vitrebond (VB)	Powder: glass powder (>95%), diphenyliodonium chloride (<2%) Liquid: copolymer of acrylic and itaconic acids (35–45%), 2-hydroxyethyl methacrylate (20–30%), water (30–40%)	3M, Saint Paul, MN, USA	Hand-mixed 1 scoop of powder with 1 drop of liquid for 15 seconds and then light-cured for 30 seconds.

BAG: bioactive glass; HEMA: 2-hydroxyethyl methacrylate; SPG: pre-reacted fluoroaluminosilicate glass.

### Preparation of powder phase of experimental RMGIC (EXP)

Sr/F-BAGs were synthesised using a sol-gel method with post-functionalisation, following a protocol described previously [18]. Briefly, an ethanol-based solution containing ethanol, deionised water and ammonium hydroxide was stirred at 600 rpm for 15 minutes, after which tetraethyl orthosilicate (TEOS) was added, and the mixture was stirred for 16–18 hours at room temperature. Silica nanoparticles were collected by centrifugation (5000 rpm, 30 minutes, 25°C) and resuspended in deionised water. Calcium nitrate, strontium nitrate and sodium fluoride were subsequently added to achieve a SiO₂:CaO:SrO:NaF molar ratio of 1.0:0.33:0.98:0.5. The particles were dried overnight at 60°C and calcined at 680°C (heating rate 3°C/min, holding time 3 hours) to remove nitrate precursors, followed by washing with ethanol.

Pre-reacted fluoroaluminosilicate glass was prepared by melting silicon dioxide, aluminium oxide, phosphorus pentoxide, calcium fluoride, zirconium dioxide and strontium carbonate in a platinum–10% rhodium crucible at 1450°C for 2 hours [19]. The molten glass was quenched in water and milled using a planetary micromill and ball-milling to obtain a median particle size of 5 µm. The glass powder was mixed with 2 wt% acid polymer liquid to form a slurry, which was spray-dried to produce spherical particles (~25 µm) of pre-reacted fluoroaluminosilicate glass (SPG)[24]. These were blended with irregular glass particles (~5 µm) at a 60:40 mass ratio to optimise filler packing.

### Preparation of the liquid phase of experimental RMGIC (EXP)

Methacrylate-functionalised polyacrylic acid was synthesised according to a previously reported method [19]. Acrylic acid and maleic acid were copolymerised in aqueous solution using potassium persulfate as an initiator and isopropanol as a chain-transfer agent at 80°C for 4 hours under nitrogen. The copolymer was purified by dialysis and freeze dried, then functionalised with glycidyl methacrylate in tetrahydrofuran using pyridine as a catalyst and butylated hydroxytoluene as an inhibitor.

The liquid phase of EXP consisted of the acid copolymer (50 wt%), 2-hydroxyethyl methacrylate (HEMA; 5 wt%) and water (45 wt%), supplemented with tartaric acid (2 pph), camphorquinone (0.7 pph) and N,N’-dimethylaminoethyl methacrylate (1.4 pph).

### Preparation of experimental RMGIC specimens

The experimental RMGIC was mixed using a powder-to-liquid ratio of 1.5:1 by weight. The powder phase of each material, including uncured TC paste, was gold-sputter-coated and examined using scanning electron microscopy (SEM) with energy-dispersive X-ray spectroscopy (EDX) to assess surface morphology and elemental composition.

### Mineral precipitation on demineralised dentine

Thirty human third molars were obtained from the Postgraduate Dental Clinic, Faculty of Dentistry, Thammasat University, following approval by the Human Research Ethics Committee of Thammasat University (Science; COE No. 028/2568). Informed consent was waived as patient identification was not required. Teeth exhibiting caries, cracks or developmental defects were excluded. Coronal dentine sections (~2 mm thick) were prepared and ultrasonically cleaned. Specimens were demineralised using 17% Ethylenediaminetetraacetic acid (EDTA) at 37°C for 6 hours to produce partially demineralised dentine, following a modified protocol from previous studies [21, 25, 26].

**Figure 2:**
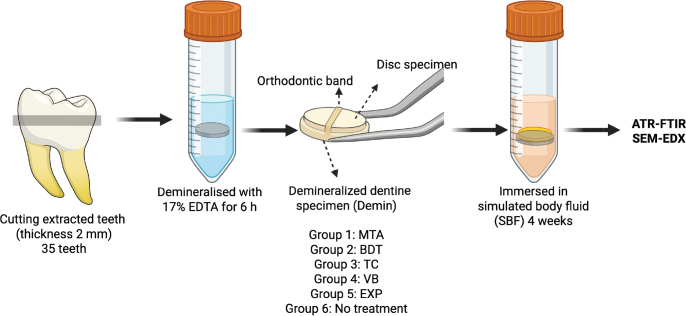
Experimental setup for the dentine remineralisation assessment. Created in BioRender. Panpisut, P. (2026) https://BioRender.com/w35d26e.

Disc specimens (10 mm diameter × 1 mm thickness) of each material were fabricated and allowed to set for 24 hours. Demineralised dentine specimens were attached to material discs (*n* = 5 per group) using orthodontic bands (Figures 1 and 2). The groups were MTA, BDT, TC, VB and EXP, with untreated demineralised dentine serving as the control. Specimens were immersed in simulated body fluid prepared according to BS ISO 23317:2014 [27] at 37°C (Figure 2).

The precipitation of minerals in the dentine was quantified using ATR-FTIR spectroscopy (Nicolet iS5, Thermo Fisher Scientific, Waltham, MA, USA) at a resolution of 8 cm^-1^ with 16 scans from 700 to 4000 cm^-1^. The mineral-to-collagen ratio was calculated from the phosphate peak at 1024 cm^-1^ (Abs_₁₀₂₄_) [28] and the Amide I peak at 1636 cm^-1^ (Abs_₁₆₃₆_) [29–31]. The percentage change of the mineral-to-collagen ratio (%) was determined using Equation 1.


$$\begin{array}{l} \mathrm{Mineral}–\mathrm{to}–\mathrm{collagen ratio}=\\ 100\;x\;\frac{\mathrm{Ratio at}\;4\mathrm{weeks}–\mathrm{Ratio at demin}}{\mathrm{Ratio at demin}} \end{array}$$ Equation 1

The ATR-FTIR assessment was performed initially, after demineralisation and after immersion in simulated body fluid (SBF) for 1, 2 and 4 weeks. At each time point, the specimens were blotted dry, and the FTIR spectra of the dentine surface were recorded. The specimens were then placed in a fresh solution. After 4 weeks, the representative dentine and material specimens from each group were sputter-coated with gold (23 mA, 45 seconds; Q150R, Quorum Technologies, East Sussex, UK) to assess surface morphology and elemental composition. The test was performed using SEM (JSM 7800F, JOEL, Tokyo, Japan) with EDX (X-Max 20, Oxford Instruments, Abingdon, UK) at 10 kV.

### pH changes

Disc specimens of each material (10 mm in diameter and 1 mm in thickness) were prepared (*n* = 3). They were immersed in 5 mL of deionised water. The measurement was performed using a pH meter (Orien Versastor Pro, Thermos Fisher Scientific, Waltham, MA, USA). The pH calibration was performed at pH 4, 7 and 10. The pH of the solution was measured at 3 hours, 24 hours, 5 days, 7 days and 28 days of immersion [26].

### Elemental release

Disc specimens (10 mm in diameter and 1 mm in thickness) were prepared (*n* = 3). They were immersed in 5 mL of deionised water at 37°C for 4 weeks. The elemental analysis was performed using inductively coupled plasma optical emission spectroscopy (ICP-OES, Optima 8300, PerkinElmer, Waltham, MA, USA) to determine the released concentrations (mg/L) of sodium (Na), aluminium (Al), silica (Si), phosphorus (P), calcium (Ca) and strontium (Sr). The calibration was performed using the Environmental Standard 26 environmental standard components (CPAchem, Bulgaria). Data analysis was performed using Syngistix TM for ICP software version 2.0 (PerkinElmer, Waltham, MA, USA). The detection limits for each element were as follows: 0.5–10 mg/L for Na (589.592 nm), 0.1–50 mg/L for Si (251.611 nm), 0.1–10 mg/L for both Ca (317.933 nm) and P (213.617 nm) and 0.1–50 mg/L for Sr (460.733 nm).

### In vitro cytotoxicity

The test was conducted following the protocol used in the previous study [19]. Disc specimens of each material (6 mm in diameter and 0.6 mm in thickness) were prepared (*n* = 4). The specimens were sterilised using UV light for 60 minutes on both the top and bottom sides. Human dental pulp stem cells (hDPSCs) were purchased from Lonza (Lonza Group AG, Basel, Switzerland), so ethical approval was not required. The cells were maintained in dental pulp stem cell basal medium (DPSCBM, Lonza Group AG, Basel, Switzerland) containing 10% dental pulp stem cell growth supplement (DPSCGS, Lonza Group AG, Basel, Switzerland) at 37°C in a 5% CO₂ humidified atmosphere.

For the extract preparation, sterilised disc specimens were immersed in 200 µL of fresh DPSCBM in a 48-well plate and incubated at 37°C in a 5% CO₂ humidified atmosphere for 3 days. After the incubation period, the specimens were removed, and the conditioned medium (extracts) from each material group was collected and stored at 4°C until use. For the cytotoxicity assessment, 100 µL of the conditioned medium was mixed with 100 µL of fresh DPSCBM (diluted two-fold) and transferred to 96-well plates. The hDPSCs at Passage 4 were then seeded at a density of 2.5 × 10³ cells/well into the 96-well plates containing the diluted conditioned medium. Plain culture medium (DPSCBM with 10% DPSCGS) without material extracts was used as the negative control. On day 1, the conditioned medium in the test groups was replaced with fresh diluted conditioned medium, while the control group received fresh plain medium. The cells were cultured for a total of 3 days at 37°C in a 5% CO₂-humidified atmosphere.

After 3 days of culture, an MTT viability assay was performed. The culture medium was removed, and cells were incubated with 3-(4,5-dimethylthiazol-2-yl)-2,5-diphenyltetrazolium bromide (MTT) solution (Sigma-Aldrich, St. Louis, MO, USA) at 37°C for 4 hours. The MTT solution was then removed, and the reaction was stopped by adding dimethylsulfoxide (DMSO, Sigma-Aldrich, St. Louis, MO, USA) to dissolve the formazan crystals. The absorbance of the resulting coloured solution was measured at 570 nm using a microplate reader (Multiskan Sky, Thermo Fisher Scientific, Waltham, MA, USA) and reported as optical density (OD). Four independent experiments were performed. The results were reported as the relative cell viability (%) compared with the negative control (plain culture medium without cells) using Equation 2.


$$Relative\;cell\;vialbility=\;100\;x\;\frac{OD\;of\;the\;test\;group}{OD\;of\;the\;negative\;control}$$ Equation 2

Where OD is the optical density.

### Statistical analysis

Values were reported as mean with 95% CI or SD. The data were analysed with GraphPad Prism version 10.0 (GraphPad Software, San Diego, CA, USA). The normality was tested using the Shapiro-Wilk test. Data with normal distribution were analysed using one-way ANOVA followed by the Tukey post-hoc test. For a non-normal distribution, the data were analysed using Kruskal-Wallis followed by the Dunn test. Additionally, a post-hoc power analysis was performed for the remineralisation test using G*power 3.1.9.6 (Heinrich-Heine-Universität Düsseldorf, Düsseldorf, Germany) [32]. The results confirmed that a sample size of *n* = 5 per group provided a power of 0.98 at an alpha level of 0.05 for one-way ANOVA (calculated effect size = 0.74).

## Results

### Analysis of material composition

**Figure 3:**
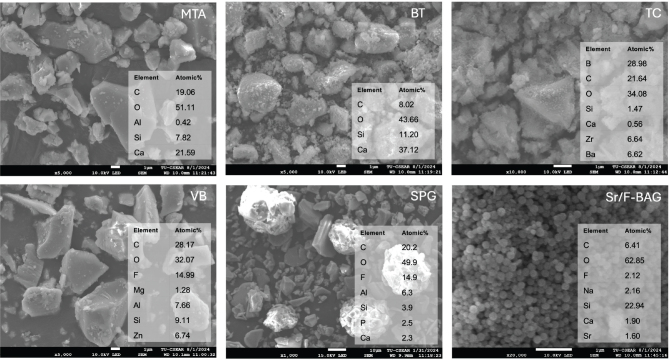
Elemental composition of the powder phase of each material determined by SEM-EDX analysis. SEM-EDX: scanning electron microscopy-energy-dispersive X-ray spectroscopy.

SEM and EDX analyses of the powder phases are presented in Figure 3. The powders of MTA, BDT and TC were primarily composed of calcium (Ca) and silicon (Si). Fluorine (F) was detected in the powder phase of VB and EXP. Strontium (Sr) was identified in the Sr/F-BAGs.

### Mineral precipitation on demineralised dentine

**Figure 4:**
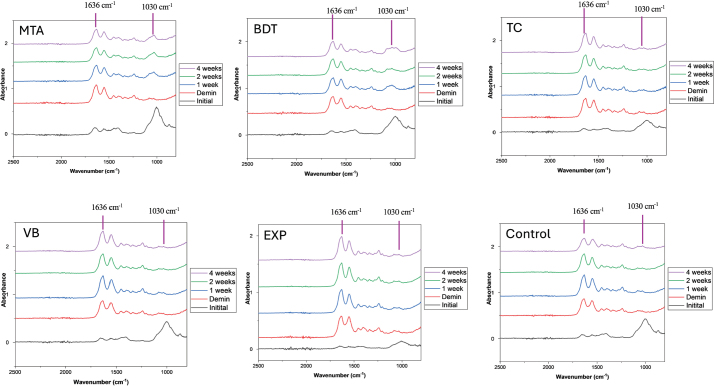
FTIR spectra of dentine specimens following demineralisation and after a demineralisation/remineralisation cycle over 4 weeks. The mineral-to-collagen ratio was calculated from the height of the FTIR phosphate peak at approximately 1024–1030 cm^-1^ relative to the amide I peak at approximately 1636 cm^-1^.

**Figure 5:**
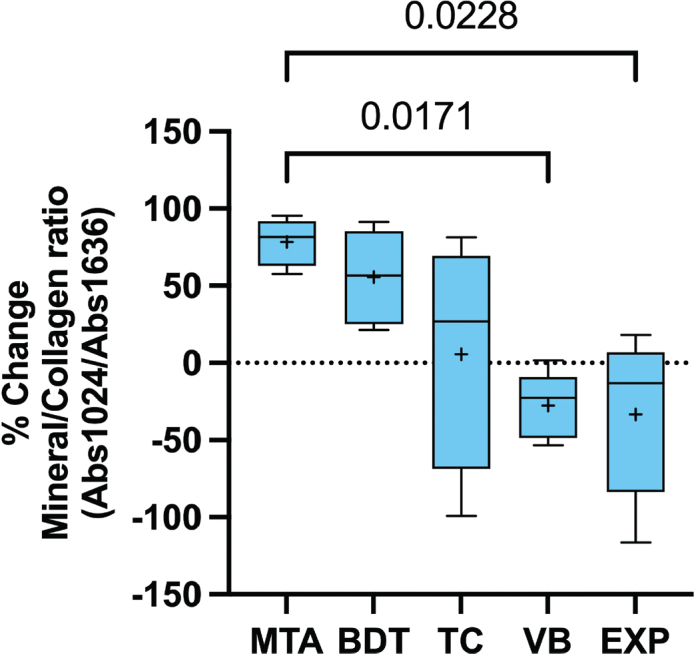
Percentage recovery of the mineral-to-collagen ratio after 4 weeks of immersion in simulated body fluid. Whiskers represent the minimum and maximum values; box limits indicate the first and third quartiles. The central line and ‘+’ symbol denote the median and mean, respectively. Lines indicate statistically significant differences (*p* < 0.05; *n* = 5). Data from the control group are not shown to improve comparison among experimental materials.

An increase in the FTIR phosphate peak associated with hydroxyapatite formation was more evident in dentine specimens attached to MTA and BDT than in those attached to other materials (Figure 4). The highest percentage recovery of the mineral-to-collagen ratio was observed with MTA (78.3 ± 15.3%), followed by BDT (55.5 ± 31.0%) and TC (5.7 ± 74.0%) (Figure 5). In contrast, VB and EXP exhibited negative percentage changes in the mineral-to-collagen ratio (−27.7 ± 21.6% and −33.3 ± 52.8%, respectively). The result from the control group was −53.5 ± 200.0%. The mineral-to-collagen ratio of MTA was significantly higher than that of VB and EXP (*p* < 0.05). No significant differences were detected between EXP and BDT (*p* = 0.293), TC (*p* > 0.99) or VB (*p* > 0.99).

**Figure 6:**
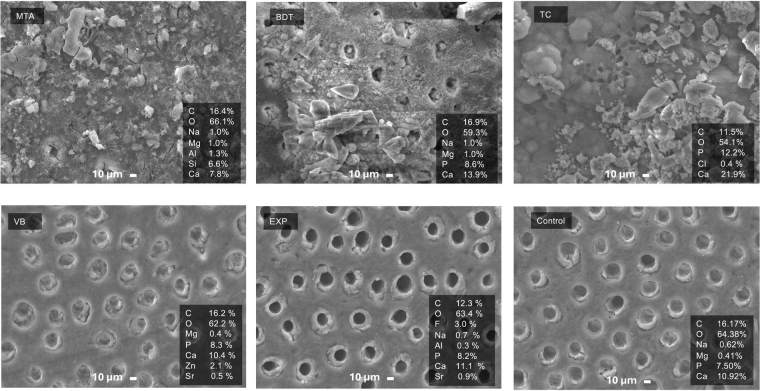
SEM images of dentine surfaces after 4 weeks of immersion in simulated body fluid. The control group consisted of demineralised dentine without material treatment. SEM: scanning electron microscopy.

SEM analysis of the dentine surfaces after 4 weeks of immersion in simulated body fluid demonstrated substantial mineral precipitation in the MTA, BDT and TC groups, whereas minimal or no mineral deposition was observed in the VB, EXP and control groups (Figure 6). Mineral precipitates occluding dentinal tubules were clearly evident in the Ca-Si cement groups.

**Figure 7:**
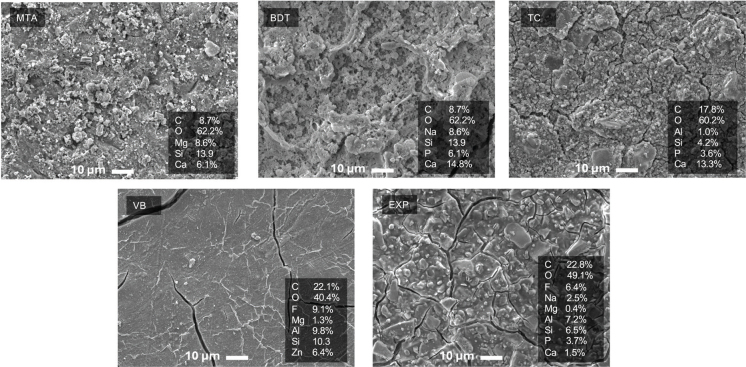
SEM images of material surfaces after 4 weeks of immersion in simulated body fluid. SEM: scanning electron microscopy.

SEM examination of the material surfaces revealed dense mineral accumulation on MTA, BDT and TC, while VB and EXP exhibited minimal surface precipitation (Figure 7). EDX analysis of MTA and BDT surfaces showed Ca and Si predominantly, whereas EXP and VB surfaces exhibited Ca, P and F.

### pH change

**Figure 8:**
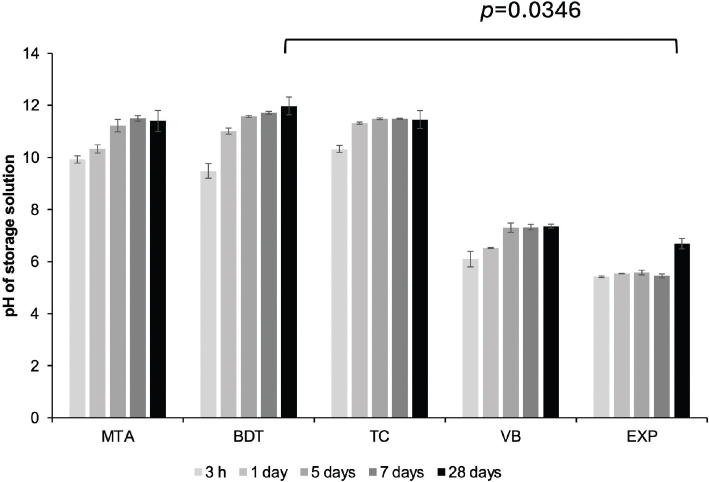
Changes in pH of the storage solution (deionised water) following immersion of materials for up to 28 days. Lines indicate statistically significant differences at 28 days (*p* < 0.05). Error bars represent SD (*n* = 3).

MTA, BDT and TC produced a rapid increase in pH, reaching approximately pH 10 within 3 hours of immersion (Figure 8). The pH of these materials gradually increased to approximately pH 11 and remained alkaline throughout the 28-day period. In contrast, VB and EXP exhibited lower initial pH values (pH ~5 to 6). The pH of VB increased to approximately 7 at 5 and 28 days, while EXP reached neutral pH only at 28 days. At 28 days, BDT exhibited the highest pH value (12.0 ± 0.4), which was significantly higher than that of EXP (6.7 ± 0.2; *p* = 0.0346).

### Elemental release

The elemental release profiles of all materials are summarised in Table 2. VB exhibited the highest sodium (Na) release (125.6 ppm), followed by EXP (15.2 ppm), which was significantly higher than that of MTA, BDT and TC (*p* < 0.05). Aluminium (Al) release was highest with VB, while BDT exhibited Al concentrations below the detection limit.

**Table 2:** Elemental release (ppm) from the tested materials after 4 weeks of immersion in deionised water, presented as mean and standard deviation. Identical letters within the same column indicate p < 0.05.

Material/Element	Na	Al	Si	P	Ca	Sr
**MTA**	1.00 (0.11)^c^	1.47 (0.39)^a,b^	0.51 (0.32)^d^	NA	125.31 (16.70)^a,b^	4.02 (0.83)^c^
**BDT**	1.67 (0.12)^c^	NA	0.14 (0.06)^d^	NA	679.56 (66.77)^a^	1.11 (0.28)^d^
**TC**	0.69 (0.08)^c^	1.30 (0.20)^b^	20.52 (1.88)^c^	0.25 (0.02)^a^	114.12 (12.84)^a,b^	53.20 (1.67)^a^
**VB**	125.62 (3.15)^a^	22.42 (2.33)^a^	39.12 (0.61)^b^	0.34 (0.11)^a^	NA	0.38 (0.04)^d^
**EXP**	15.17 (1.65)^b^	2.17 (0.61)^a,b^	70.04 (3.36)^a^	1.98 (0.17)^a^	16.64 (8.21)^b^	25.77 (1.39)^b^

NA indicates concentrations below the detection limit of the instrument.

EXP demonstrated the highest silicon (Si) release (70.0 ppm), whereas BDT showed the lowest Si release (0.1 ppm). Phosphorus (P) release was detected in EXP (2.0 ppm), VB (0.3 ppm) and TC (0.3 ppm). Calcium (Ca) release was highest with BDT (679.6 ppm), followed by MTA (125.3 ppm), TC (114.1 ppm) and EXP (16.6 ppm). Strontium (Sr) release was highest with TC (53.2 ppm), followed by EXP (25.8 ppm).

### In vitro cytotoxicity

**Figure 9:**
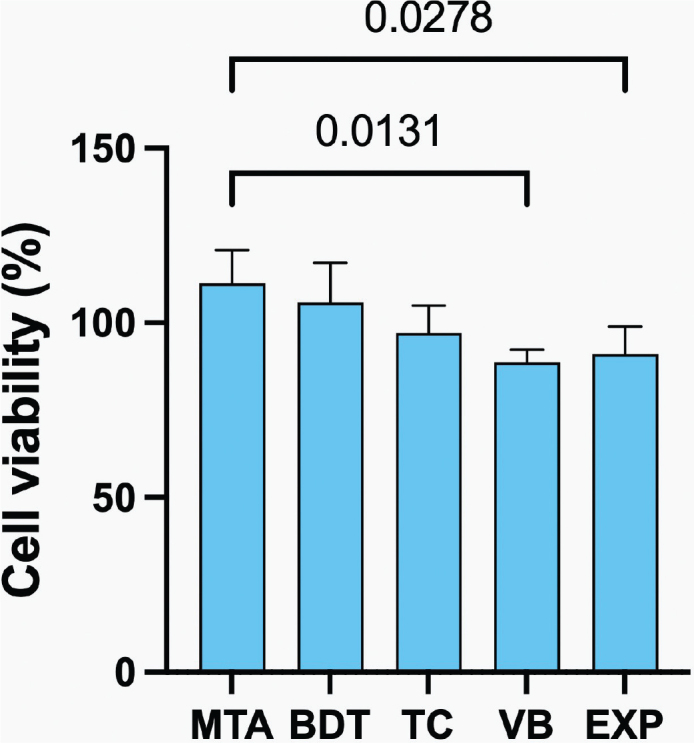
Cell viability of human dental pulp stem cells after exposure to material extracts. Lines indicate statistically significant differences (*p* < 0.05). Error bars represent 95% CI (*n* = 4).

The highest cell viability was observed in hDPSCs exposed to MTA extracts (111 ± 10%), whereas the lowest viability was observed with VB (89 ± 4%) (Figure 9). Cell viability values for BDT (106 ± 11%), TC (97 ± 8%), EXP (91 ± 8%) and VB were comparable (*p* > 0.05). However, MTA exhibited significantly higher cell viability than VB (*p* = 0.0127) and EXP (*p* = 0.0268). All materials demonstrated cell viability exceeding 70%.

## Discussion

This study evaluated dentine remineralisation, pH changes, elemental release and *in vitro* cytotoxicity of an experimental RMGIC containing Sr/F-BAGs (EXP). Although EXP demonstrated inferior mineral precipitation and pH elevation compared with Ca-Si cements, it promoted the release of multiple essential ions and exhibited satisfactory cytocompatibility comparable to commercial materials. Therefore, the null hypothesis was partially accepted.

Ca‑Si cements, including MTA, BDT and TC, demonstrated substantial mineral precipitation on demineralised dentine, consistent with previous studies [33, 34]. When Ca-Si cements interact with simulated body fluid containing phosphate ions, apatite precipitates form and coat both the material and dentine surfaces. In addition, the release of hydroxyl ions creates a highly alkaline environment, while the formation of a silicate network further enhances the remineralisation of demineralised dentine [35]. Surface apatite formation has also been suggested to promote a tight seal between the material and the dentine surface, potentially contributing to improved interfacial stability [33]. Although the percentage recovery of the mineral-to-collagen ratio among Ca-Si cements was comparable, the present findings align with previous reports indicating that resin-free Ca-Si cements, such as MTA and BDT, exhibit more rapid and greater remineralisation than TC [35].

The lower remineralising effect of EXP compared with Ca-Si cements may be primarily attributed to differences in setting reactions. Ca-Si cements set mainly through a hydration process, resulting in the release of Ca^2+^ and OH^–^ ions, which subsequently react with available phosphate ions to facilitate apatite nucleation and precipitation on demineralised dentine [36, 37]. In contrast, EXP relies on an acid–base reaction characteristic of glass ionomer systems, leading to the release of ions such as F^–^, Sr^2+^ and Ca^2+^ that participate in ion-exchange interactions at the tooth–material interface [38]. Although this mechanism may create conditions favourable for mineral deposition [7], the inherently acidic environment associated with RMGICs may limit precipitation reactions [39]. This may subsequently result in reduced remineralising efficacy despite the incorporation of Sr/F-BAGs.

Another factor that may have contributed to the limited remineralisation observed with EXP is the relatively short immersion period of 4 weeks. Previous studies have reported significant increases in mineral density and recovery of caries-affected dentine following treatment with RMGICs or Ca-Si cements after longer immersion periods of approximately 3–6 months [40, 41]. Therefore, future investigations should evaluate the remineralisation potential of EXP over extended durations. In addition, remineralisation in the present study was assessed using ATR-FTIR spectroscopy, which primarily reflects surface mineral changes and may not fully capture the depth or volume of mineral deposition.

Ca-Si cement extracts demonstrated higher cytocompatibility with human dental pulp stem cells than RMGIC extracts. This finding may be attributed to the absence of unreacted low-molecular-weight methacrylate monomers, such as HEMA, in Ca-Si cements [42]. In contrast, the acidic environment associated with EXP and VB may be less favourable for cell viability [43]. The low pH associated with EXP may additionally result in lower cell viability of the experimental RMGIC. Furthermore, Ca-Si cements release high concentrations of calcium and silicon ions, which have been shown to activate the mitogen-activated protein kinase (MAPK) signalling pathway, thereby promoting cell viability and proliferation [44]. Nevertheless, all materials exhibited cell viability exceeding 70%, indicating a low risk of cytotoxicity according to ISO 10993-5:2009 standards [45].

Several limitations of this study should be acknowledged. First, the experiments were conducted *in vitro*, and the clinical relevance of the findings should therefore be interpreted with caution. Second, the chemical demineralisation model used may not fully replicate the complex biological and microbiological challenges of natural caries progression [46]. Future studies employing biofilm-based demineralisation models may better simulate clinical conditions. The lesions produced using such models more closely resemble natural caries-affected dentine in terms of surface morphology and collagen degradation [47]. The alternative techniques, such as micro-computed tomography [41], transverse microradiography [48], or polarisation-sensitive optical coherence tomography [49], may be needed in future work to provide a more comprehensive assessment of the depth and distribution of remineralisation. Future work should also include the experimental RMGIC without Sr/F-BAGs to examine the beneficial effect of the reactive fillers.

## Conclusion

The experimental RMGIC incorporating Sr/F-BAGs demonstrated inferior remineralisation and pH-elevating capacity compared with Ca-Si cements. However, the experimental material promoted the release of multiple essential ions while maintaining acceptable *in vitro* cytocompatibility.

## Data Availability

Raw data are available upon request to the corresponding author.
